# WWOX-Related Epileptic Encephalopathy (WOREE Syndrome): Clinical Case Study and Literature Review

**DOI:** 10.3390/cimb48050449

**Published:** 2026-04-25

**Authors:** Annamaria Sapuppo, Roberta Rizzo, Gaia Fusto, Roberta Rocca, Vincenzo Sortino, Xena Giada Pappalardo, Martino Ruggieri, Raffaele Falsaperla

**Affiliations:** 1 Unit of Pediatrics and Pediatric Emergency Department, Azienda Ospedaliero-Universitaria Policlinico “Rodolico-San Marco”, San Marco Hospital, 95123 Catania, Italy; 2 Department of Biomedical and Biotechnological Sciences (BIOMETEC), University of Catania, 95123 Catania, Italy; rizzo.roberta@studium.unict.it (R.R.); gaia.fusto@studium.unict.it (G.F.); xena.pappalardo@irib.cnr.it (X.G.P.); 3 Postgraduate Training Program in Pediatrics, Department of Clinical and Experimental Medicine, University of Catania, 95123 Catania, Italy; uni367297@studium.unict.it; 4 Unit of Catania, Institute for Research and Biomedical Innovation (IRIB), National Council of Research, 95126 Catania, Italy; vincenzo.sortino@unikorestudent.it; 5 PhD Program in Innovative Technologies in Biomedical Sciences, University Kore of Enna, 94100 Enna, Italy; 6 Unit of Clinical Pediatrics and Unit of Rare Disease AOU “Policlinico”, PO “G. Rodolico”, University of Catania, 95123 Catania, Italy; m.ruggieri@unict.it; 7 Department of Medical Science-Pediatrics, University of Ferrara, 44124 Ferrara, Italy; raffaele.falsaperla@unife.it

**Keywords:** autosomal recessive spinocerebellar ataxia 12 (SCAR12; MIM: 614322), infantile epileptic encephalopathy 28 (IEE28; MIM: 616211), WW domain-containing oxidoreductase (*WWOX*) gene (MIM: 605131), *WWOX*-related epileptic encephalopathy (WOREE) syndrome, whole exome sequencing (WES)

## Abstract

The WW domain-containing oxidoreductase (*WWOX*) gene, well-known as a tumor suppressor, also has a crucial role as a transcription factor in the developing brain. The bi-allelic loss of the *WWOX* gene causes a condition characterized by drug-resistant epilepsy, developmental delay, and neurological impairments, often resulting in mortality within the first year of life, known as *WWOX*-related epileptic encephalopathy (WOREE) syndrome (MIM: 616211). Whole Exome Sequencing (WES) analysis was performed on a female patient who died within three months of birth and was diagnosed with microcephaly, severe early-onset refractory seizures, and drug-resistant epileptic encephalopathy. WES revealed a 38 kb CNV deletion spanning *WWOX* exons 6–7, and a known frameshift variant in exon 8, impairing a highly clinically significant region of the encoded protein. Clinical and genetic features of reported WOREE patients with *WWOX* gene deletions similar to our patient were analyzed. Our case highlights the clinical heterogeneity of *WWOX* variants in WOREE syndrome and expands the spectrum of reported compound heterozygous deletions. Further research needs to elucidate *WWOX* pathophysiology and improve diagnostic and therapeutic strategies.

## 1. Introduction

The WW domain-containing oxidoreductase (*WWOX*) gene (MIM: 605131) is located within the fragile site *FRA16D* on chromosome 16q23.3–q24.1, a well-known hotspot of genomic instability associated with both germline and somatic copy number variations (CNVs) [1,2]. Germline variants in *WWOX* are rare and are primarily associated with severe neurodevelopmental disorders, whereas somatic alterations have been implicated in cancer predisposition [2]. Widely recognized as a tumor suppressor gene, *WWOX* encodes a 414-amino acid enzyme belonging to the short-chain dehydrogenase/reductase (SDR/ADH) family, which also exerts multifunctional roles, including transcriptional regulation and modulation of key cellular pathways such as differentiation, apoptosis, and steroid metabolism [1]. Although less extensively studied in the brain, *WWOX* plays a crucial role in central nervous system development, contributing to neuronal differentiation, migration, and proliferation [3]. It is expressed in neurons, oligodendrocytes, and astrocytes, where it functions as a scaffold protein through its WW domain, enabling interactions with multiple partners, including cytoskeletal components and proteins involved in intracellular trafficking and microtubule-based transport [3]. Biallelic pathogenic variants in *WWOX* are associated with autosomal recessive disorders, including spinocerebellar ataxia type 12 (SCAR12; MIM: 614322) and infantile epileptic encephalopathy 28 (IEE28; MIM: 616211) [2]. These conditions typically present with neurological manifestations such as epilepsy, developmental delay, and motor impairment [2]. Experimental animal models have provided important insights into *WWOX* deficiency, demonstrating phenotypes resembling human disease. In particular, *WWOX* knockout (KO) mice recapitulate key features including epilepsy, structural brain abnormalities, learning deficits, severe metabolic disturbances, and early postnatal lethality within weeks after birth [4]. To date, 88 disease-causing variants in *WWOX* are reported in the Human Gene Mutation Database (HGMD; www.hgmd.cf.ac.uk/), highlighting distinct genotype–phenotype correlations: (i) null genotypes, associated with absent psychomotor development, lack of spontaneous motility and eye contact from birth, drug-resistant epilepsy in early infancy, retinal degeneration, premature death, and acquired microcephaly; (ii) compound heterozygous genotypes involving one null allele and one missense variant, associated with a milder encephalopathy characterized by developmental delay and increased survival; and (iii) biallelic hypomorphic missense variants, associated with spinocerebellar ataxia type 12 (SCAR12) [5]. Homozygous and compound heterozygous variants in *WWOX* have also been linked to WOREE (*WWOX*-related epileptic encephalopathy) syndrome (www.wwox.org), which is characterized by severe early-onset epileptic encephalopathy with seizures resistant to antiseizure medications, along with diverse structural brain abnormalities, including impaired myelination, corpus callosum thickening, white matter abnormalities, and/or cerebral atrophy [6,7]. Affected individuals also present with developmental delay, severe psychomotor impairment, spasticity with hyperreflexia, hypokinesia, and an inability to walk or communicate verbally. Microcephaly has also been reported [8,9], and most affected individuals die within the first year of life [2]. Despite recent advances, the full phenotypic spectrum and precise genotype–phenotype correlations of *WWOX*-related disorders remain incompletely understood and are still under active investigation.

In this study, we report a female patient who died within a few months of life and presented with microcephaly and severe early-onset refractory seizures consistent with drug-resistant epileptic encephalopathy. Trio-based whole-exome sequencing (WES) identified two variants in the proband: a 38 kb CNV deletion encompassing *WWOX* exons 6–7 and a known frameshift variant c.1043del (p.Phe348Serfs*57) in exon 8 (ClinVar ID: 421407). This study aims to contribute to the understanding of the rare *WWOX*-associated disease spectrum by integrating the clinical and genetic features of this case.

## 2. Materials and Methods

### 2.1. Ethical Compliance

The study was conducted ethically in accordance with the World Medical Association Declaration of Helsinki and approved by the Ethics Committee of the University of Catania, Italy (Ethical Committee Catania 1 Clinical Registration n. 210/2023/PO; approval date, 1 October 2023). Written informed consent was obtained from the parents of the patient for publication of the details of their medical case and any accompanying images.

### 2.2. Genetic Testing Information

The genomic DNA of the proband was extracted from a buccal swab to perform the clinical WES using the Blueprint Genetics® Whole Exome Plus Test(Blueprint Genetics^®^, Helsinki, Finland, version 3, 27 February 2023), that consists of sequence analysis of all protein-coding genes in the genome for the proband, coupled with Whole Exome Deletion/Duplication (CNV) Analysis. This test targets all protein-coding exons, exon-intron boundaries (±20 bps) and selected noncoding, deep intronic variants, single-nucleotide variants and small insertions and deletions (INDELs) up to 220 bps and CNVs defined as single-exon or larger deletions and duplications. This test should not be used for the detection of repeat expansion disorders, balanced translocations, complex inversions and low-level mosaicism. The analysis of the Whole Exome Plus Test is primarily focused on established disease genes that have been previously associated with genetic disorders. The genes with known clinical association include those curated by Blueprint Genetics® (BpG) and included in BpG diagnostic panels (>4140 genes). These genes are supplemented with genes included in The Clinical Genomics Database (>4320 genes) and the Developmental Disorders Genotype-Phenotype Database (DD2GP) (>2190 genes). The total number of genes that are considered clinically associated in the Whole Exome Plus analysis is >4780 (and the number is constantly updated). If analysis of exome variants in previously established disease genes is inconclusive, exome variant data are also analyzed for variants that are not located within known clinically associated genes but have properties that make them candidates for potentially disease-causing variants (please see: https://www.blueprintgenetics.com/tests/whole-exome-sequencing/ (accessed on 27 February 2023)). The performance metrics of sequencing reported for the nuclear genome included a median coverage of 148 and 99.56% of target bases covered at >20×. For the mitochondrial genome, the median coverage was 27,652, with 100% of target bases covered at >1000×. The annotation of clinical relevance of identified variants was assessed by considering the relevant literature and databases such as 1000 Genomes Project, ClinVar, HGMD Professional, Database of Genomic Variants, ExAC, gnomAD and DECIPHER. For the interpretation of mtDNA variants, specific databases, including, e.g., Mitomap, HmtVar and 1000G, were used. The pathogenicity potential of variants was also evaluated using in silico variant prediction tools, such as SIFT (version 0.7.0, http://sift-dna.org/), PolyPhen (version 2, https://genetics.bwh.harvard.edu/pph2/) and MutationTaster (version 2021, https://www.genecascade.org/MutationTaster2021/; accessed on 4 March 2023), which were used to assist with the variant classification established by the American College of Medical Genetics and Genomics (ACMG) guideline 2015 [10]. The study was approved by the NICU ethics committee, and the proband’s parents provided written consent in accordance with the Declaration of Helsinki.

### 2.3. Literature Review

A systematic literature review was conducted to identify published case reports describing individuals with *WWOX* variants affecting exons 6–8. The search was performed across multiple electronic databases, including PubMed, Embase, and Scopus, covering studies published from January 2000 to March 2026. The search strategy combined controlled vocabulary and free-text terms, including “*WWOX*,” “*WWOX* gene,” “exon 6,” “exon 7,” “exon 8,” “epileptic encephalopathy,” “WOREE syndrome,” and “case report,” using appropriate Boolean operators. The initial search yielded a total of 200 records. After removal of duplicates, 150 unique records were screened based on titles and abstracts. Of these, 50 articles were excluded for not meeting the inclusion criteria. Full-text assessment was conducted for 30 studies, of which 70 were excluded due to insufficient clinical or genetic detail. Ultimately, 20 case reports were included in the final analysis. Inclusion criteria comprised: (i) original case reports or case series describing individuals with *WWOX* variants specifically involving exons 6–8, and (ii) availability of sufficient clinical and genetic data. Exclusion criteria included studies lacking key clinical and genetic information, specifically sex, ethnicity, presence of microcephaly, detailed seizure characteristics (including age at onset and frequency), evidence of drug resistance, electroencephalographic (EEG) and brain magnetic resonance imaging (MRI) findings, survival data, and precise genetic variant description. Only articles published in English were considered.

## 3. Results

### 3.1. Case Presentation

**Figure 1:**
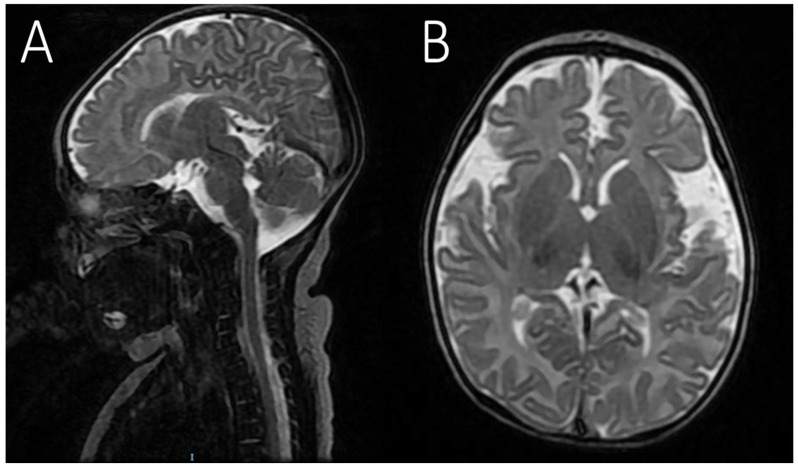
Brain MRI of our patient. (**A**) Sagittal T2 sequence; (**B**) Axial T2 sequence showing incomplete myelination, normal for age (10 days). No other abnormalities involving the cerebral, cerebellar, and brainstem parenchyma.

The patient, a female infant, was admitted to the neonatal intensive care unit (NICU) due to seizures a few hours after birth. She was born after an uneventful pregnancy and spontaneous vaginal delivery at full term. The birth weight was 3.100 g (−0.88 SDS—Standard Deviation Score), the length was 50 cm (−0.2 SDS), and the head circumference was 32 cm (−2.07 SDS), consistent with microcephaly. Apgar scores were 9 and 10 after 1 and 5 min, respectively. The familiar medical history was unremarkable. Seizures, characterized by body stiffening, horizontal nystagmus, eyelid myoclonus and epileptic spasms, began shortly after birth and were accompanied by vomiting. There was no significant family history or dysmorphic features noted upon examination. The infant was started on levetiracetam for seizure management. The first EEG was normal, but due to the persistence of seizures, treatment with levetiracetam as an ASM was initiated. Neurological examination revealed several abnormalities, including lack of gaze fixation with horizontal nystagmus, axial hypotonia with spastic hypertonia of the limbs, reduced general movements, and reduced patellar reflexes. At 8 days of life, she presented with multi-daily asymmetric tonic seizures with eye deviation to the left and left arm hyperextension. A second EEG performed during sleep showed multifocal interictal epileptiform discharges (IED) with a focus on the bilateral centro-temporal areas. The ictal EEG revealed spikes on the right occipital derivations. Based on the electrical pattern and the persistence of critical episodes, carbamazepine was added as an adjunctive ASM. At this stage, the differential diagnosis included early infantile epileptic encephalopathy, neonatal encephalopathy secondary to hypoxic–ischemic encephalopathy, and encephalopathy due to structural brain abnormalities. The EEG pattern strongly indicated early infantile epileptic encephalopathy. Blood tests for metabolic disorders were performed, resulting within normal limits. Brain MRI at 10 days of age was unremarkable. Unfortunately, the infant passed away from cardiac arrest after three months of age due to pulmonary infections and respiratory failure (Figure 1).

### 3.2. Mutational Analysis

**Figure 2:**
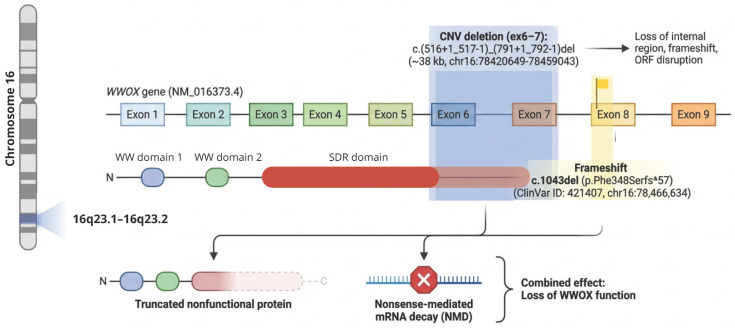
Schematic representation of the *WWOX* (NM_016373.4) gene and pathogenic variants identified in the proband. The genomic organization of *WWOX* on chromosome 16 is illustrated, including exon structure and protein domains (WW1, WW2, and SDR). The 38 kb CNV deletion c.(516+1_517-1)_(791+1_792-1)del (chr16:78420649–78459043; hg19), spanning exons 6–7, is represented in blue, while the frameshift variant c.1043del (p.Phe348Serfs*57) (ClinVar ID: 421407) is represented in yellow. Both variants are predicted to result in the loss of *WWOX* function through the generation of a truncated, nonfunctional protein and/or mRNA degradation.

WES analysis (hg19) identified two heterozygous pathogenic variants of the *WWOX* gene (NM_016373.4), a CNV deletion and a frameshift variant, illustrated in Figure 2.

The CNV deletion, also confirmed by digital polymerase chain reaction (PCR), is a novel rearrangement not found in Decipher database v.21.11 or other databases (Table 1). No additional genetic findings were reported. Analysis of asymptomatic parental samples would have been required for the inheritance pattern determination of variants, but was not performed.

## 4. Discussion

*WWOX*-related disorders display marked phenotypic heterogeneity, ranging from the milder SCAR12 presentation to the severe WOREE syndrome, reflecting the nature and functional impact of the underlying variants [7]. However, the relationship between genotype and phenotype remains incompletely defined, as considerable discrepancies are observed even among patients harboring similar variants. This variability suggests that additional factors, such as genetic background, environmental influences, or the presence of modifier genes, may significantly modulate disease expression. Consequently, diagnosis and prognostic stratification remain challenging. Although WOREE syndrome typically manifests with early-onset epilepsy, growth impairment, delayed psychomotor development, and progressive microcephaly correlated with a high early mortality, not all reported cases fully recapitulate this spectrum [3]. For instance, while microcephaly is a recurrent feature, it is not consistently observed in patients with similar *WWOX* alterations in exons 2, 6, and 7, as well as with the very early onset of epileptic encephalopathy in the first days of life [8,9], including those involving comparable CNVs (Table 1). This discrepancy raises the possibility of alternative or contributory mechanisms, including undetected variants in regulatory or deep intronic regions, epigenetic factors, or additional genomic rearrangements not captured by standard analyses.

Preclinical data, such as the lde/lde rat model (lethal dwarfism with epilepsy) carrying a 13 bp deletion in exon 9 of the *WWOX* gene, support a critical role of this gene in neurodevelopment, yet they may represent an extreme phenotype that does not fully mirror the variability observed in humans [12]. Similarly, previously reported cases highlight both overlapping and divergent clinical trajectories, including differences in age at onset, survival, and associated features, further underscoring the lack of a straightforward genotype–phenotype correlation. Functionally, the *WWOX* gene would be linked to neuronal migration and, therefore, involved in the growth of cranial circumference, hence the microcephaly. However, the pathophysiological mechanism driving the development of WOREE syndrome due to the complete or partial loss of function of *WWOX* remains incompletely understood. In our patient, WES data, in fact, showed that both pathogenic variants disrupt a clinically significant region of the protein regarding the C-terminus of the ADH/SDR and the mitochondrial binding region translated by exons 6–8 of the gene (Figure 2). However, analysis of parental samples would have been necessary to determine whether the variants occurred in cis (on the same allele) or in trans (on different alleles). Compound heterozygosity of the variants (in trans) would explain the patient’s clinical presentation.

In the literature, cases carrying similar CNVs in the *WWOX* gene have not consistently reported microcephaly (Figure 1; Table 1), although evidence of microcephaly was associated with other genotypes [9,13]. Establishing a causative and contributory role of the CNV to the fatal outcome for the child’s survival is more complex. Similar rearrangements to our CNV have been documented in the ExAC (17 individuals) and gnomAD SVs v2.1 (4 individuals) control cohort databases. However, the *WWOX* exons 6–7 deletion has been previously described in trans with a *WWOX* missense variant in at least 3 individuals with WOREE syndrome [7]. In addition, out-of-frame deletion of exon 6 or in-frame deletion of exon 7 has been found in several individuals with *WWOX*-related phenotypes [7,13,14].

Currently, there are no available data or indications correlating the protein damage caused by the frameshift mutation in exon 8 with the CNV deletion in exons 6–7. In the absence of this information, the functional loss in ADH/SHR domains and mitochondria targeting sequence of the protein is considered to be plausibly deleterious. With regard to the frameshift variant, it has already been reported in trans with exon 7–8 out-of-frame deletion or in the homozygous state in at least two individuals with *WWOX*-related developmental and epileptic encephalopathy [9,15]. As highlighted in Table 1, Piard et al. report another patient who experienced early death at 6 months of age, similar to our patient (who passed away at 3 months), with epileptic encephalopathy onset at 2 days of life in both cases [7]. In addition, there are other studies describing a clinical picture characterized by very early infantile-onset epileptic encephalopathy. Mallaret et al. reported a homozygous missense variant c.139C>A, p.(Pro47Thr), within the WW domain, in four siblings with childhood-onset cerebellar ataxia, generalized tonic–clonic epilepsy, and intellectual disability [4]. They also identified a homozygous missense variant c.1114G>C, p.Gly372Arg, within the C-terminal part of *WWOX*’s dehydrogenase/reductase domain, in another family with two affected siblings exhibiting generalized tonic–clonic epilepsy, intellectual disability, and ataxia. Moreover, early death was rarely reported [7]. Abdel-Salam et al. identified, through exome sequencing, a homozygous nonsense pathogenic variant c.160G>T, p.Arg54* in a girl from a consanguineous family with severe growth retardation, microcephaly, epileptic seizures, retinopathy, and early death [8], as in our patient. Additionally, Mignot et al. identified biallelic pathogenic variants in patients with infantile epileptic encephalopathy, including deletions, truncating and missense variants, and delineated the phenotypic spectrum of *WWOX* variants [11].

In the present study, the progressive clinical deterioration of our patient, leading to death within the first 3 months of life, suggests the potential detrimental impact that the specific *WWOX* genotype might have on lifelong prognosis. Overall, the clinical course of our patient, characterized by very early-onset epileptic encephalopathy, microcephaly, and rapid progression to death, supports a severe *WWOX*-related phenotype. However, to prevent potential misinterpretation, a causal attribution of the observed clinical features to the identified variants cannot be established. A more comprehensive genomic approach and functional studies would be necessary to clarify the pathogenic mechanisms and to better delineate the contribution of potential modifier factors.

## 5. Conclusions

To date, only one additional patient has been reported carrying a genotype characterized by compound heterozygous deletion of the *WWOX* gene in WOREE syndrome [15]. Both that case and ours involve newborns who experienced seizures shortly after birth with refractoriness to ASMs. However, notable differences emerge in neuroimaging findings: in the previously reported case, brain MRI showed white matter hyperintensity and delayed myelination, whereas MRI findings in our patient were unremarkable. More broadly, the literature describes a spectrum of neuroimaging abnormalities associated with WWOX-related disorders, including hippocampal hypoplasia, corpus callosum anomalies, enlargement of subarachnoid spaces, and asymmetry of the lateral ventricles. This variability further supports a role for *WWOX* in brain development and structural homeostasis, while also highlighting the inconsistency of imaging features across patients. Despite these differences, early mortality was observed in both cases, underscoring the severe clinical course and poor prognosis commonly associated with this condition.

A key limitation of this study is its design as a single-patient case report, which inherently restricts the generalizability of the findings. This limitation is further compounded by the absence of parental genetic testing, which precludes segregation analysis and prevents the determination of the cis/trans configuration of the identified variants, thereby limiting interpretation of their inheritance pattern and pathogenicity. In addition, the use of WES entails intrinsic technical constraints, as it may fail to detect deep intronic variants, structural rearrangements, copy number variations, or low-level mosaicism that could contribute to the observed phenotype. Consequently, alternative genetic explanations, including undetected pathogenic variants or multilocus contributions, cannot be excluded. Moreover, no functional studies were performed to validate the biological impact of the identified variants. The available clinical data are also incomplete, which may limit comprehensive phenotypic characterization. Finally, given the observational nature of this report, a definitive causal relationship between the identified genetic findings and the clinical presentation cannot be established.

## Figures and Tables

**Table 1:** A focused summary of the main clinical and genetic features of our proband and WOREE patients with 6–7 exon-containing CNV variants.

Ref.	Epidemiology		Neurological Examination					Survival	Genetic Information		
	Sex	Ethnicity/Consanguinity	Microcephaly/ Seizure	Age of Onset/ Freq. of Seizures	Drug Resistance	EEG	MRI	Premature Death (<3 m)	Variant	Exon	Protein Change
[7]	F	Italian/No	No/ focal, tonic and spasms	20 d/daily	No	disorganized, multifocal and disorganized, but without epileptic discharges	CC hypoplasia, expansion of the CSF spaces	na	c.606_791del (in frame deletion)	6–7	p.Pro203_Arg264del
	F	Caucasian/No	No/ focal (hemiclonic) and generalized spasms during the course	2–3 d/daily	Yes	abnormal background activity and bitemporal paroxysmal anomalies	CC hypoplasia, cerebral atrophy	na	c.953C>T (missense)	8	p.Ser318Leu
									c.517_1056del (in frame deletion)	6–7	p.His173_Met352del
	F	French/No	No/ focal at onset (occipital: eye movements) and tonic–clonic with eye movements	2–3 d/daily	Yes	disorganized and SBA. epileptiform activity: occipital spikes	CC hypoplasia, subarachnoid space enlargement	6 m	c.606_791del (in frame deletion)	5–7	p.Pro203_Arg264del
	M	Sub-Saharan African/Yes	No/ focal seizures hemitonic/ polymorphic: focal (hemitonic) and myoclonic jerks/ perioral myoclonia	1 m/daily	Yes	disorganized and SBA. Epileptiform activity: occipital spikes	cerebral atrophy, subarachnoid spaces enlargement, CC hypoplasia, white matter hypersignal	2.25 y	c.517_1056del (in frame deletion)	6–8	p.His173_Met352del
	M	West Indian, Britain/No	No/ infantile spasms	2–3 d/daily	Yes	hypsarrhythmic pattern	thin CC, abnormal lateral ventricles	2.5 y	c.517_791del (in frame deletion)	6–7	His173Ilefs*5
	F	Britain/No	No/ infantile spasms	2–3 d/daily	Yes	modified hypsarrhythmic pattern	normal	2.5 y	c.517_1056del (in frame deletion)	6–8	p.His173_Met352del
									c.705dupG (frameshift)	7	p.His236Alafs*34
	M	French/No	No/ focal clonic l and generalized with dialeptic seizures and myoclonic seizures	2–3 d/daily	Yes	abnormal sleeping background with focal temporal spikes on initial EEG	thin CC	na	c.517_791del (in frame deletion)	6–7	p.His173Ilefs*5
	M	French/No	No/ focal and clonic at onset, then spasms and myoclonic seizures	2–3 d/daily	Yes	abnormal sleeping background with focal temporal spikes on initial EEG	CC hypoplasia, cerebral atrophy	na	c.517_791del (in frame deletion)	6–7	p.His173Ilefs*5
[11]	F	Chinese/No	No/ seizures characterized by clenched teeth, cyanosis around the lips and tetanic twitch of the limbs	15 d/daily	Yes	slow weakening of background activity observed in both hemispheres and polyspikes low-wave discharge in bilateral temporal lobes	white matter hyperintensity and delayed myelination in the brain	2.5 y	c.517_605del (frameshift)	6	p.His173AlafsTer67
									c.517_1056del (in frame deletion)	6–8	p.His173_Met352del
Present case	F	Italian/No	Yes/ body stiffening, horizontal nystagmus, eyelid myoclonus and epileptic spasms	2 d/daily	Yes	spikes on the right occipital derivations	normal	3 m	c.(516+1_517-1) _(791+1_792-1) (out of frame deletion)	6–7	p.His173AlafsTer67
									c.1043delT (frameshift)	8	p.Phe348SerfsTer57

Abbreviations: CC, corpus callosum; d, days; F, female; M, male; m, months; na, not available; SBA, slow background activity.

## Data Availability

The data presented in this study are available on request from the corresponding author. The data are not publicly available due to reasons of sensitivity.

## Abbreviations

The following abbreviations are used in this manuscript:

ADH/SDR	Short-chain Dehydrogenases/Reductases
ACMG	American College of Medical Genetics and Genomics
ASMs	Anti-Seizure Medications
bp	Base pairs
CNVs	Copy Number Variations
DD	Developmental Delay
EEG	Electroencephalogram
ExAC	Exome Aggregation Consortium
HGMD	Human Gene Mutation Database
IEE	Infantile Epileptic Encephalopathy
IEE28	Infantile Epileptic Encephalopathy 28
IED	Interictal Epileptiform Discharges
KO	Knockout
MIM	Mendelian Inheritance in Man
MRI	Magnetic Resonance Imaging
NICU	Neonatal Intensive Care Unit
PCR	Polymerase Chain Reaction
SCAR12	Autosomal Recessive Spinocerebellar Ataxia
SDS	Standard Deviation Score
VEP	Variant Effect Predictor
WES	Whole Exome Sequencing
WOREE	*WWOX*-Related Epileptic Encephalopathy
